# Personal

**Published:** 1893-02

**Authors:** 


### Personal.

---

DR. D. B. ST. JOHN ROOSA was elected President of the New York Academy of Medicine, at its annual meeting, held January 5, 1893. It is stated that the largest vote was cast on this occasion that has been known in the history of the Academy. In electing Dr. Roosa to this high office, the Academy has reflected quite as much honor upon itself as it has conferred upon the able and erudite physician whom it has chosen to be its chief executive officer for the next two years.

---

Dr. G. Frank Lydston, of Chicago, was tendered a dinner by the Detroit Medical Library Association, at the Hotel Cadillac, on Monday evening, January 9, 1893. After the dinner, Dr. Lydston delivered a lecture in the hall of the society, entitled Etiology of Prostatic Hypertrophy. A large number of the profession of Detroit, including its most prominent members, was present.

